# Immunological heterogeneity informs estimation of the durability of vaccine protection

**DOI:** 10.1098/rsif.2022.0070

**Published:** 2022-05-25

**Authors:** Matthieu Domenech de Cellès, Anabelle Wong, Laura Andrea Barrero Guevara, Pejman Rohani

**Affiliations:** ^1^ Infectious Disease Epidemiology group, Max Planck Institute for Infection Biology, 10117 Berlin, Germany; ^2^ Institute of Public Health, Charité—Universitätsmedizin Berlin, 10117 Berlin, Germany; ^3^ Odum School of Ecology, University of Georgia, Athens, GA 30602, USA; ^4^ Department of Infectious Diseases, University of Georgia, Athens, GA 30602, USA; ^5^ Center for Influenza Disease & Emergence Research (CIDER), University of Georgia, Athens, GA 30602, USA

**Keywords:** SARS-CoV-2, COVID-19, vaccines, duration of immunity, heterogeneity, mathematical modelling

## Abstract

Deciphering the properties of vaccines against an emerging pathogen is essential for optimizing immunization strategies. Early after vaccine roll-out, however, uncertainties about vaccine immunity raise the question of how much time is needed to estimate these properties, particularly the durability of vaccine protection. Here we designed a simulation study, based on a generic transmission model of vaccination, to simulate the impact of a breadth of vaccines with different mean (range: 10 months–2 years) and variability (coefficient of variation range: 50–100%) of the duration of protection. Focusing on the dynamics of SARS-CoV-2 in the year after start of mass immunization in Germany as a case study, we then assessed how confidently the duration of protection could be estimated under a range of epidemiological scenarios. We found that lower mean and higher heterogeneity facilitated estimation of the duration of vaccine protection. Across the vaccines tested, rapid waning and high heterogeneity permitted complete identification of the duration of protection; by contrast, slow waning and low heterogeneity allowed only estimation of the fraction of vaccinees with rapid loss of immunity. These findings suggest that limited epidemiological data can inform the duration of vaccine immunity. More generally, they highlight the need to carefully consider immunological heterogeneity when designing transmission models to evaluate vaccines.

## 1. Introduction

As vaccines against an emerging pathogen become available, assessing their properties is essential for epidemic forecasting and for optimizing immunization strategies. Key among those properties is the duration of vaccine protection, whose distribution can schematically be decomposed into the population mean and the inter-individual variability. A large body of epidemiological theory indicates that the mean duration of vaccine protection predicts essential metrics of long-term control, like critical vaccination coverage and vaccine impact [1–3]. Although less studied [4–6], the variability in the duration of protection may be equally important to predict the long-term dynamics of an emerging pathogen after mass immunization, perhaps even more so because heterogeneity appears to be a defining feature of vaccine immunity [7]. Elucidating the duration of vaccine protection can be achieved by identifying immune correlates of protection, a central tenet of vaccinology [8]. However, the search for such correlates may be complicated by the intricacies of vaccine immunity, often characterized by substantial heterogeneity and multiple memory components with different kinetics of decay (as in the case of COVID-19 vaccines [9]). Alternatively, the rate of waning vaccine effectiveness can be estimated from longitudinal cohort studies conducted after vaccine roll-out. However, the interpretation of such studies may be complex, as it depends on the study design [10] and on the mode of vaccine protection [11,12].

As a complement to immunological and epidemiological vaccine studies, mathematical models of transmission and vaccination—which allow translating hypotheses about individual-level mechanisms of vaccine protection into population-level dynamics—may be helpful to unravel vaccine immunity [13–16]. Earlier applications demonstrated the ability of such models to accurately estimate relevant attributes of various vaccines while capturing both their direct and indirect effects on infection dynamics [17–20]. However, as the epidemiology of an emerging pathogen may rapidly change (e.g. because of the emergence of new variants, as exemplified by SARS-CoV-2 [21]), a practical question is how much longitudinal data are needed to estimate the duration of vaccine protection using such models, given the inevitable trade-off between fast and accurate estimation. A critical element controlling this trade-off is the number of breakthrough cases in vaccinated individuals developing over time, as more events of vaccine failure will help inform the duration of vaccine protection. This number is itself determined by the specific force of infection and vaccination coverage in the study population, in addition to general vaccine properties like the duration of protection. Importantly, the mean and the variability of this duration jointly modulate the fraction of vaccinees losing immunity and thus the number of breakthrough cases during a given time period. From a theoretical perspective, therefore, it can be hypothesized that both the mean and the variability of the duration of vaccine protection can affect the time required for reliable estimation.

Here, we designed a simulation study to determine how reliably the duration of vaccine immunity against an emerging pathogen could be estimated with age-specific case report incidence data. We developed a generic population-based model of vaccination, applied—as a case study—to represent the dynamics of SARS-CoV-2 in the year after the start of mass immunization against COVID-19 in Germany. Emphatically, we used this model as a general framework—as opposed to a specific investigation of COVID-19 vaccines—to examine how the amount of data and the characteristics of the distribution of vaccine protection durability affected estimation performance. We find that limited epidemiological data can provide information on the duration of vaccine protection. In addition, we show that higher vaccine heterogeneity facilitates estimation of this duration. Altogether, these results highlight the need to pay careful attention to immunological heterogeneity when designing transmission models to estimate or predict the impact of vaccines.

## 2. Methods

### 2.1. Study population and study period

In this simulation study, we considered Germany as our study population and we simulated a study period of at most 1 yr following the introduction of COVID-19 vaccines (i.e. time zero in the model was set to the start time of vaccination). As vaccination against COVID-19 started in late 2020/early 2021 in Germany, the study period approximately covered year 2021 and initial conditions were assumed to represent the epidemiological situation at the beginning of that year. The endpoints considered were case reports of clinical and subclinical SARS-CoV-2 infections.

### 2.2. Model formulation and parametrization

We used a previously described deterministic model of SARS-CoV-2 transmission, empirically estimated with data in six countries (China, Italy, Japan, Singapore, Canada, and South Korea) during the first wave (December 2019–March 2020) of COVID-19 [22]. Briefly, the model is an extension of the standard Susceptible–Exposed–Infected–Recovered (SEIR) model, stratified according to age (*I* = 8 age groups considered here: 0–9, 10–19, … , 60–69 and ≥70 yr) and according to type of infection, either subclinical (defined as asymptomatic or paucisymptomatic) or clinical. To add biological realism, the latent and the infectious periods were assumed Gamma-distributed with a shape parameter of 2 and a mean of 1/*σ* = 3 days and 1/*γ* = 5 days, respectively [22]. The age-specific susceptibility to SARS-CoV-2 infection (denoted by *u_i_*_=1,…,*I*_) and clinical fraction (*y_i_*_=1,…,*I*_) were fixed from the estimates in [22] and are displayed in electronic supplementary material, figure S2. We assumed that subclinical infections were *θ* = 50% as transmissible as clinical infections, the value fixed in [22] and also consistent with estimates during the first wave in China [23]. The rates of contacts between age groups were fixed using data from the POLYMOD study in the UK [24], corrected for reciprocity with 2019 age-specific demographic data in Germany (electronic supplementary material, figure S3). Although Germany was also part of the POLYMOD study, to keep our model general we did not use the corresponding data because they were found to differ markedly (with fewer contacts among children and adolescents) from those in other European countries, according to a previous modelling study of pertussis [25].

To model changes in SARS-CoV-2 transmission over the year following the start of vaccination, we used a flexible time-varying function *β*(*t*):

$$\log\beta (t)=\log\beta_{0}+\beta_{1}t+\beta_{2}\sin\omega t+\beta_{3}\sin2\omega t+\beta_{4}\sin3\omega t,$$

where *ω* = 2*π*/365 day^−1^ and *β*(0) = *β*_0_. Although arbitrary, this function was chosen to allow multiple (at most three) peaks over the year, as observed in time series of the effective reproduction number during year 2020 in European countries. The exponential trend was used to model the gradual spread of SARS-CoV-2 variants of concern, in particular the Alpha and Delta variants estimated to be 29 (95% CI: 24–33)% and 97 (95% CI: 76–117)% more transmissible than non-variants [26]. As of June 2021, the Alpha variant predominated in Germany [26], and we therefore tested scenarios ranging from absent to complete replacement with the Delta variant by the end of the study period (that is, $1.24\leq e^{365\;\beta_{1}}\leq 2.17$). At start of vaccination, we assumed an initial effective reproduction number *R*_e_(0) = 1.1 [27]; the corresponding value of *β*_0_ was calculated by equating *R*_e_(0) to the leading eigenvalue of the next-generation matrix [22]:

$${\mathrm{NGM}}_{ij}=\beta_{0}u_{i}\frac{c_{ij}}{\gamma }[y_{j}+\theta (1-y_{j})](1-r_{i}(0))\frac{N_{i}}{N_{j}},$$

where *r_i_*(0) is the initial fraction recovered and *N_i_* the population size in age group *i*. The other transmission parameters *β_i_*_=1,…,4_ were estimated from the simulated data, as explained below. In every age group, we assumed an initial prevalence of past infections of 10% [28], of exposed infections of 10^−4^ and of active infections of 10^−4^. We supposed all initial conditions to be known with reasonable accuracy, for example from epidemiological studies (such as seroprevalence studies) or from hindcasts of epidemiological models around the time of vaccine introduction.

Next, we extended the model described above to incorporate vaccination against COVID-19. Specifically, we assumed that COVID-19 vaccines conferred imperfect immunity against clinical infections (vaccine effectiveness against clinical infections: VE_C_ = 1 − *ε*_C_, where *ε*_C_ is the leakiness to clinical infections) and against subclinical infections (vaccine effectiveness against subclinical infections: VE_S_ = 1 − *ε*_S_). Vaccine protection was assumed leaky—as opposed to ‘all-or-nothing’ [3]—based on the observations from phase 1/2 clinical trials, which detected a neutralizing antibody response in all vaccine recipients after a booster dose [29–32]. Finally, vaccine protection was assumed to wane over time, at rate *α*. Using the method of stages [33], the distribution of the duration of protection was varied by subdividing the vaccinated class *V* into *n*_V_ sub-classes. The resulting distribution *D*_V_ was Gamma with shape parameter *n*_V_, mean $E(D_{V})=1/\alpha$, and coefficient of variation $\mathrm{CV}(D_{V})=\sqrt{V(D_{V})}/E(D_{V})=1/\sqrt{n_{V}}$. Vaccination was assumed to vary over time and to occur continuously, at rate *v_i_*(*t*) in age group *i*. Although applied to COVID-19 vaccines in the present case study, this vaccine model is largely generic and was used in previous modelling studies of other vaccine-preventable infections [12,19,20,34].

Given these assumptions, the model was given by the following set of differential equations (see full list of model parameters in table 1 and model schematic in electronic supplementary material, figure S1):

$$\begin{array}{rl} {\dot{V}}_{i,1} & =v_{i}(t)S_{i}-n_{V}\alpha V_{i,1}-[\epsilon_{C}y_{i}+\epsilon_{S}(1-y_{i})]\;\lambda_{i}\;(t)V_{i,1},\\ {\dot{V}}_{i,j=2,\ldots ,n_{V}} & =n_{V}\alpha (V_{i,j-1}-V_{i,j})-[\epsilon_{C}y_{i}+\epsilon_{S}(1-y_{i})]\;\lambda_{i}\;(t)V_{i,j},\\ {\dot{S}}_{i} & =-[v_{i}\;(t)+\lambda_{i}\;(t)]S_{i},\\ {\dot{S}}_{V,i} & =n_{V}\alpha V_{i,n_{V}}-\lambda_{i}(t)S_{V,i},\\ {\dot{E}}_{C,i,1} & =y_{i}\lambda_{i}\;(t)\;(S_{i}+S_{V,i}+\epsilon_{C}V_{i})-2\sigma E_{C,i,1},\\ {\dot{E}}_{C,i,2} & =2\sigma (E_{C,i,1}-E_{C,i,2}),\\ {\dot{I}}_{C,i,1} & =2\sigma E_{C,i,2}-2\gamma I_{C,i,1},\\ {\dot{I}}_{C,i,2} & =2\gamma \;(I_{C,i,1}-I_{C,i,2}),\\ {\dot{E}}_{S,i,1} & =(1-y_{i})\;\lambda_{i}\;(t)(S_{i}+S_{V,i}+\epsilon_{S}V_{i})-2\sigma E_{S,i,1},\\ {\dot{E}}_{S,i,2} & =2\sigma (E_{S,i,1}-E_{S,i,2}),\\ {\dot{I}}_{S,i,1} & =2\sigma E_{S,i,2}-2\gamma I_{S,i,1},\\ {\dot{I}}_{S,i,2} & =2\gamma \;(I_{S,i,1}-I_{S,i,2})\\ \mathrm{and}\;\;{\dot{R}}_{i} & =2\gamma \;(I_{C,i,2}+I_{S,i,2}), \end{array}$$

where $V_{i}=\sum_{j}V_{i,j}$ is the total number of individuals vaccinated and

$$\lambda_{i}(t)=\beta (t)u_{i}\underset{j}{\sum }c_{ij}\frac{I_{C,j,1}+I_{C,j,2}+\theta (I_{S,j,1}+I_{S,j,2})}{N_{j}}$$

is the (*per capita*) force of infection in age group *i*.

**Table 1.** List of model parameters. Parameters marked with a star * were estimated from the simulated data. SA: sensitivity analysis; VE: vaccine effectiveness; CV: coefficient of variation.

parameter(s)	symbol	fixed value or range of fixed values	source/comment
*vaccination model*			
vaccination rate	*v_i_*(*t*)	electronic supplementary material, figure S5	[37,38]
VE against clinical infections	1 − *ε*_C_	0.95	[41,42] SA (electronic supplementary material, figure S8): 60%
VE against subclinical infections	1 − *ε*_S_	0.90	[43] SA (electronic supplementary material, figure S8): 50%
mean duration of vaccine protection*	E(*D*_V_) = 1/*α*	0.85, 2 years	assumption. SA (electronic supplementary material, figures S9–S10): 5 years
variability in duration of vaccine protection*	$\mathrm{CV}(D_{V})=1/\sqrt{n_{V}}$	0.50, 0.71, 1.00	assumption
*transmission model*			
average latent period	1/*σ*	3 days	[22]
average infectious period	1/*γ*	5 days	[22]
susceptibility to infection	*u_i_*	electronic supplementary material, figure S2	[22]
clinical fraction	*y_i_*	electronic supplementary material, figure S2	[22]
relative infectiousness of subclinical infections	*θ*	0.5	[22] SA (electronic supplementary material, figure S11): estimated
age-specific contact rates	*c_ij_*	electronic supplementary material, figure S3	[24,48]
initial reproduction number	*R*_e_(0)	1.1	[27]
initial transmission coefficient	*β*_0_	0.045	calculated so that *R_e_*(0) = 1.1
trend in transmission rate*	*β*_1_	(6–21) × 10^−4^ per day	[26], variable across scenarios
seasonal transmission parameters*	*β*_2,3,4_	(–0.3 to 0.3)	variable across scenarios
initial fractions exposed	*E_i_*(0)	10^−4^	assumption
initial fractions infected	*I_i_*(0)	10^−4^	assumption
initial fractions recovered	*R_i_*(0)	0.1	seroprevalence studies [28]
*observation model*			
probability of reporting clinical infections	*ρ*_C_	0.5	[35]
probability of reporting subclinical infections	*ρ*_S_	0.05	assumption (cf. [36] and main text). SA (electronic supplementary material, figure S11): estimated
over-dispersion in case reporting*	*k*_C_	0.04	CV of observation model ≈20%

To complete the model specification and to generate the simulated data, we used a negative binomial observation model, an extension of the Poisson model with over-dispersion allowing for extra variability in the mean reporting probability. Specifically, we assumed that *ρ*_C_ = 50% of clinical infections were reported to the surveillance system, in keeping with previous estimates during March–June 2020 in Germany [35]. The reporting fidelity of subclinical infections (denoted by *ρ*_S_) is less well known, but can be evaluated by comparing the burden estimated from case reports and sero-epidemiological surveys. Such comparison indicated an overall (i.e. including clinical and subclinical infections) reporting probability of 15–20% in Germany [36], suggesting low reporting of subclinical infections. Here we assumed that 95% of subclinical infections were unreported (*ρ*_S_ = 0.05). The over-dispersion in case reporting was fixed to *k* = 0.04 (approximate coefficient of variation of the observation model: 20%) for the simulations and subsequently estimated from the simulated data. Given the intensive surveillance of COVID-19, we assumed that the data consisted of daily case reports (without reporting delay and overall, with no extra information on clinical or subclinical infection) in every age group. Although delays in case reporting are inevitable in practice, they can be accurately modelled from other data sources (e.g. line data with dates of symptom onset and of notification, available from a sample of reported cases) and easily incorporated into transmission models for added realism. Hence, including such delays would not affect the inference problem considered here, and we ignored them for simplicity.

### 2.3. Vaccine properties and epidemiological scenarios considered

In accordance with vaccination strategies defined in Germany and in most other European countries [37], vaccination was assumed continuous and staggered, starting with individuals ≥70 yr, followed by 60–69 yr, 20–59 yr and 10–19 yr. Based on vaccination coverage data observed until 1 September 2021 (plotted in electronic supplementary material, figure S5) [38], we assumed that 85% of ≥70 yr, 85% of 60–69 yr, 65% of 20–59 yr and 50% of 10–19 yr would get vaccinated 0–240, 60–240, 90–240 and 150–365 days after start of vaccination, respectively. Based on available immunological evidence [9,39,40], we assumed a mean duration of protection of 0.85 yr (rapid waning) or 2 yr (slow waning), and we contrasted three different levels of variability: 50% (low heterogeneity), 71% (intermediate heterogeneity) and 100% (high heterogeneity). The lower bound of the mean duration was also chosen because it resulted in an identical fraction of vaccinees losing immunity within the study period of 1 yr, irrespective of heterogeneity (electronic supplementary material, figure S4C). Other vaccine attributes were assumed known and were fixed based on the results of clinical trials and early epidemiological studies of mRNA vaccines: vaccine effectiveness of 95% against clinical infections [41,42] and of 90% against subclinical infections [43].

To generate different hypotheses about the spread of SARS-CoV-2 in the year following start of vaccination, we randomly sampled values for the trend parameters (*β*_1,…,4_). To add realism, we selected 10 parameter sets that resulted in 10 simulated datasets (henceforth referred to as epidemiological scenarios) meeting the following two criteria: (1) overall cumulative incidence of total cases less than or equal to 10% and (2) no peak in overall case reports during summer. These criteria were chosen to be consistent with the *a priori* hypothesis of persistent, but low circulation of SARS-CoV-2 in the year following start of vaccination.

### 2.4. Parameter estimation

For every scenario, the following parameters were estimated from the data: rate of waning vaccine protection (*α*), transmission parameters (*β*_1,…,4_) and over-dispersion in case reporting (*k*_C_). Estimation was conducted using maximum-likelihood estimation via trajectory matching [44]. In a first analysis, we assumed that the variability in the duration of vaccine protection was known (i.e. *n*_V_ was fixed to its true value), and we calculated the profile log-likelihood to estimate the maximum-likelihood estimate and the 99% confidence interval (CI) of the average duration of vaccine protection (1/*α*) [45]. We evaluated the profile log-likelihood on a grid of 100 values in the range 0.05–50 years, uniformly distributed on a logarithmic scale. For every grid point, estimation was conducted 10 times, with starting parameter values sampled over broad ranges using a Latin hypercube design. In a second analysis, we assumed that the variability in the duration of protection was unknown, and we estimated both the mean and the coefficient of variation of the duration of protection. The estimation proceeded similarly, except that the profile log-likelihood was evaluated on a grid of 100 values of the fraction with short-term immunity (here defined as the proportion of individuals losing immunity within 1 year after vaccination, denoted by *p*_1_), uniformly arranged in the interval 0.005–0.995. We focused on this fraction in the second analysis, because, even though all the distributions considered had the same mean, their overall shape differed markedly (see electronic supplementary material, figure S4). All likelihood profiles were inspected visually and subsequently smoothed using generalized additive models (GAMs), with automatic estimation of the degree of smoothing. An approximate 99% confidence interval (for 1/*α* or *p*_1_) was calculated as the range of GAM-predicted 99% lower bounds of smoothed parameter values within *χ_p_*_=0.99,d.f.=1_/2 ≈ 3.32 log-likelihood units of the maximum log-likelihood.

### 2.5. Assessment of estimation performance

To assess estimation performance, we quantified both the accuracy and the precision of the estimates of the average duration of vaccine protection. Specifically, we measured estimation accuracy by either the bias or the mean absolute bias (defined as the mean absolute difference between the estimated values and the true value). To evaluate estimation precision, we calculated the mean 99% CI width, averaged across epidemiological scenarios. Furthermore, we examined the range of lower and upper 99% CI bounds. If the lower (upper) confidence bound equalled the lower (upper) limit of the estimation interval (0.05–50 years) for a given epidemiological scenario, the corresponding parameter estimates were considered practically non-identifiable for decreasing (increasing) parameter values [45].

### 2.6. Numerical implementation

All models were implemented, simulated and estimated using the pomp package [44,46], operating in R v. 3.6.3 [47]. The socialmixr package was used to calculate the contact matrix [48], based on the data from the POLYMOD survey included therein [24]. The renv package was used to keep track of all packages' versions and to increase reproducibility [49]. All R programming codes are freely available from Edmond, the Open Data Repository of the Max Planck Society: https://doi.org/10.17617/3.AWRHE8.

## 3. Results

### 3.1. Estimation results for slow-waning vaccines

**Figure 1.**
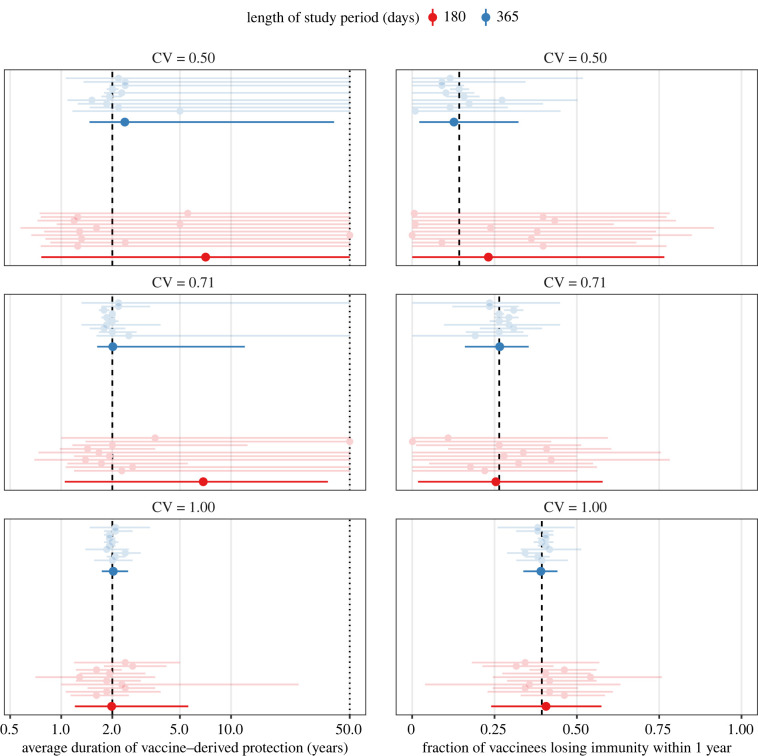
Estimates of the average duration of protection (left panels) and of the fraction with short-term immunity (right panels), assuming known variability in the duration of protection conferred by low-waning COVID-19 vaccines. The black dashed lines indicate the true values used in numerical simulations, corresponding to a mean duration of protection of 2 yr (full distribution displayed in electronic supplementary material, figure S4A). For each of 10 simulations (ordered by increasing simulation number from bottom to top and displayed in electronic supplementary material, figure S6), light points (intervals) represent the maximum-likelihood estimate (99% confidence interval), calculated using the profile log-likelihood. Solid points (intervals) represent the corresponding quantities, averaged across simulations. In the left panels, the *x*-axis values are log_10_-transformed for visual clarity; the dotted line indicates the maximal value tested for profile likelihood evaluation and the assumed limit for practical idenfiability. CV: coefficient of variation, quantifying the variability in the duration of vaccine protection.

We first present the results for slow-waning vaccines, assumed to confer a mean duration of protection of 2 yr. The resulting simulations are displayed in electronic supplementary material, figure S6, and the estimates of the average duration of protection—under the assumption that the degree of variability was known *a priori*—in figure 1 (left panels). For low (CV = 0.5, bottom panel) and intermediate (CV = 0.71, middle panel) variability in the duration of protection, we found that estimation based on six months of daily data was typically inaccurate and imprecise. In most scenarios (7/10 and 10/10 for intermediate and low variabilities), the limited amount of data resulted in practical non-identifiabilities, such that an upper confidence bound could not be derived. However, a lower confidence bound could be derived in all scenarios tested (range of lower 99% CI bounds: 0.6–1.4 yr). By contrast, estimates were markedly more accurate (mean absolute bias: 0.4 yr) and precise (mean 99% CI width: 4.4 yr) for high variability in the duration of vaccine protection (CV = 1.00, lower panel). As expected, increasing the length of the study period to 1 yr improved estimation in all scenarios tested. For high variability, precise and accurate estimates of the mean duration of protection could consistently be derived (mean absolute bias: 0.1 yr, mean 99% CI width: 0.7 yr). Although non-identifiability persisted for lower levels of variability in some scenarios (2/10 for intermediate variability and 8/10 for low variability), a precise lower confidence bound could be consistently calculated (range of lower bounds: 1.1–1.9 yr). Hence, we found evidence that, all else being equal, higher variability facilitated estimation of the mean duration of vaccine protection.

These results suggest that, when vaccine heterogeneity is low, information present in one year of epidemiological data permits reliable estimation of only the lower, but not the upper confidence limit for the mean duration of protection. However, such information is highly relevant from a public health perspective, because the lower confidence limit relates to the degree of early loss of vaccine protection. It can therefore caution public health actors in decision-making when it comes to infection prevention and surveillance. To illustrate, we present in figure 1 (right panels) the estimates of the fraction with short-term immunity. Despite the practical non-identifiabilities reported above, we found that relatively accurate and precise estimation of this key metric was possible with only 1 yr of data (mean absolute bias ranging from 1.4% for high variability to 5.1% for low variability). Importantly, despite identical mean fixed in all simulations, the fraction with short-term immunity increased with the variability in the duration of vaccine protection. These findings emphasize the need to pay careful attention to the full distribution of the duration of vaccine protection to understand the post-vaccination dynamics of SARS-CoV-2, and more generally of an emerging pathogen.

**Figure 2.**
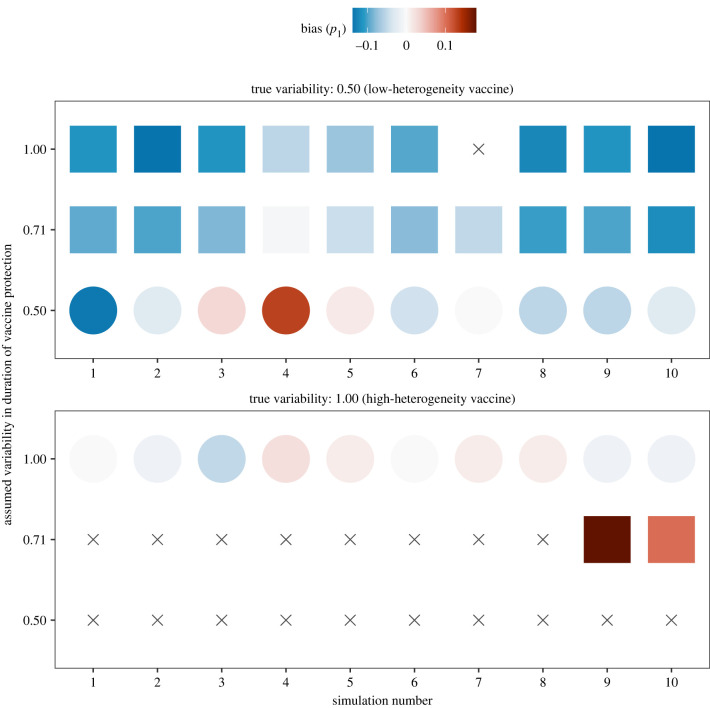
Estimates of the fraction with short-term immunity, assuming unknown variability in the duration of protection conferred by low-waning COVID-19 vaccines. The colours of the filled circles or squares indicate the bias in the estimated fraction with short-term immunity (*p*_1_), for different epidemiological scenarios (*x*-axis) and different levels of variability in the duration of vaccine protection (CV, *y*-axis). As in figure 1, the mean duration of vaccine protection was fixed to 2 yr in all scenarios tested; circles (squares) indicate simulations for which the variability was correctly (incorrectly) specified. Grey crosses indicate simulations more than $1/2\chi_{\;p=0.99,d.f.=1}^{2}\approx 3.32$ log-likelihood units away from the maximum log-likelihood and therefore not in the 99% confidence interval for the corresponding scenario.

In practice, because of early uncertainties about vaccine immunity, quantitative estimates of the heterogeneity of protection may not be available. Next, we therefore proceeded to estimate both the mean of, and the variability in, the duration of vaccine protection from our set of epidemiological scenarios. In simulations of vaccines with low heterogeneity (CV = 0.50, true fraction with short-term immunity of 14%, figure 2, top panel), we found that almost all models resulted in an equally good fit to the data, such that the degree of variability could not be identified. For mis-specified levels of variability, the fraction with short-term immunity was systematically underestimated (mean bias of –7.7% for intermediate variability and of –10.6% for high variability). In simulations of vaccines with high heterogeneity (CV = 1.00, true fraction with short-term immunity of 39%, figure 2, bottom panel), we found that more information on the degree of variability was present in the simulated data. Specifically, the hypothesis of low variability could be rejected in all scenarios and the true variability recovered in 8. In the other 2 scenarios, mis-specifying the level of variability led to over-estimating the true fraction with short-term immunity (bias values: 10 and 18%). These results confirm that higher variability helps estimate the full duration of vaccine protection.

### 3.2. Estimation results for rapid-waning vaccines

**Figure 3.**
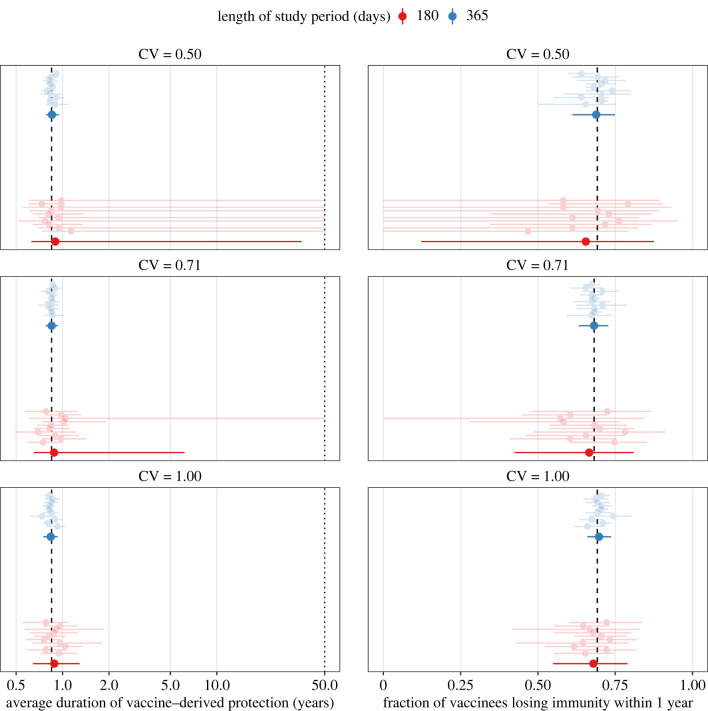
Estimates of the average duration of protection (left panels) and of the fraction with short-term immunity (right panels), assuming known variability in the duration of protection conferred by high-waning COVID-19 vaccines. The black dashed lines indicate the true values used in numerical simulations, corresponding to a mean duration of protection of 0.85 yr, or approximately 10 months (full distribution displayed in electronic supplementary material, figure S4B). For each of 10 simulations (ordered by increasing simulation number from bottom to top and displayed in electronic supplementary material, figure S7), light points (intervals) represent the maximum-likelihood estimate (99% confidence interval), calculated using the profile log-likelihood. Solid points (intervals) represent the corresponding quantities, averaged across simulations. In the left panels, the *x*-axis values are log_10_-transformed for visual clarity; the dotted line indicates the maximal value tested for profile likelihood evaluation and the assumed limit for practical idenfiability. CV: coefficient of variation, quantifying the variability in the duration of vaccine protection.

**Figure 4.**
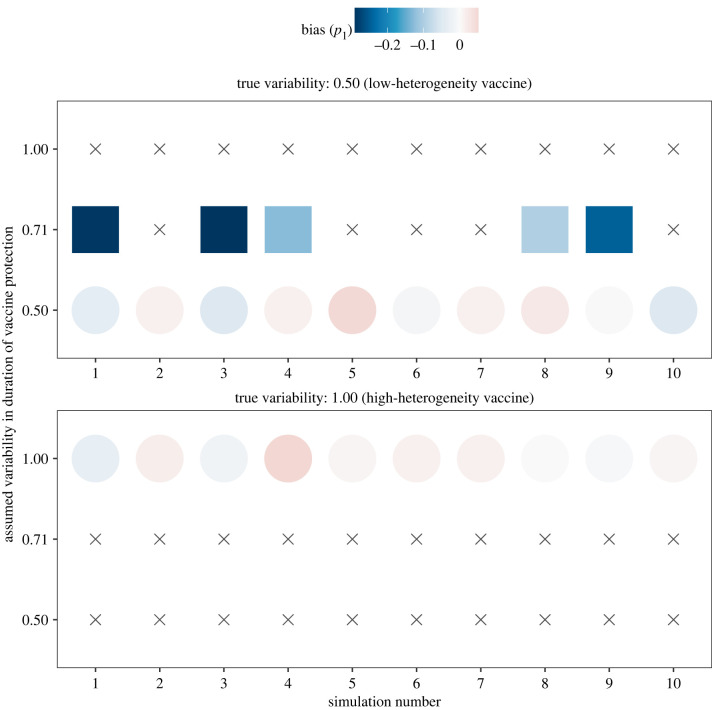
Estimates of the fraction with short-term immunity, assuming unknown variability in the duration of protection conferred by high-waning covid vaccines. The colours of the filled circles or squares indicate the bias in the estimated fraction with short-term immunity (*p*_1_), for different epidemiological scenarios (*x*-axis) and different levels of variability in the duration of vaccine protection (CV, *y*-axis). As in figure 3, the mean duration of vaccine protection was fixed to 0.85 yr in all scenarios tested; circles (squares) indicate simulations for which the variability was correctly (incorrectly) specified. Grey crosses indicate simulations more than $1/2\chi_{\;p=0.99,d.f.=1}^{2}\approx 3.32$ log-likelihood units away from the maximum log-likelihood and therefore not in the 99% confidence interval for the corresponding scenario.

To interpret the results above, one may hypothesize that higher vaccine heterogeneity facilitates estimation only to the extent that it increases the fraction of individuals losing immunity during the study period of 1 yr (figure 1*b* and electronic supplementary material, figure S4C). To test the validity of this hypothesis, we next considered rapid-waning vaccines, with mean duration of protection fixed to 0.85 yr, or approximately 10 months. This particular value was chosen so that the fraction with short-term immunity was almost identical across the levels of vaccine heterogeneity (*p*_1_ = 0.68 for intermediate variability, *p*_1_ = 0.69 for low and high variability; figure 3*b* and electronic supplementary material, figure S4C). Assuming the degree of variability to be known *a priori*, estimates of the average duration of protection were consistently accurate with six months of daily data (mean absolute bias of 0.1 yr for all levels of variability; figure 3*a*). The estimates were also generally precise for vaccines with high (mean 99% CI width: 0.7 yr) and intermediate (mean 99% CI width: 5.5 yr, 1/10 scenario with non-identifiable upper bound) heterogeneity. By contrast, low-heterogeneity vaccines typically resulted in imprecise estimation (7/10 scenarios with non-identifiable upper bound). With 1 yr of daily data, estimation performance became excellent, irrespective of vaccine heterogeneity (mean absolute bias: less than 0.05 yr, mean 99% CI width: 0.2 yr for every level of variability). This amount of data also allowed estimating not only the mean of, but also the variability in, the duration of protection in all scenarios of high-heterogeneity vaccines (figure 4, bottom panel). For low-heterogeneity vaccines (figure 4, top panel), the hypothesis of high heterogeneity could be rejected in all scenarios, but the true variability recovered in only 5. In the other 5 scenarios, in keeping with the results for low-waning vaccines, the fraction with short-term immunity was underestimated when vaccine heterogeneity was over-estimated (bias range: –29% to –10%). Hence, despite equal loss of immunity (as measured by the mean duration of protection or the fraction with short-term immunity) across these scenarios, higher variability again helped estimate the duration of vaccine protection. These results show that vaccine heterogeneity is an independent predictor of estimation performance.

### 3.3. Sensitivity analyses

To test the robustness of our results, we conducted three sensitivity analyses. First, to take into account the range of efficacy estimates from clinical trials of COVID-19 vaccines [41,42,50,51], we tested slow-waning vaccines with mean duration of protection of 2 yr but a lower effectiveness of 60% against clinical infections and of 50% against subclinical infections. We found our main results to hold in this case, with broadly similar patterns in parameter identifiability as vaccine heterogeneity was varied (electronic supplementary material, figure S8). However, estimation precision substantially improved, particularly for vaccines with intermediate (mean 99% CI width decreasing from 10.4 yr to 0.6 yr) and low (mean 99% CI width decreasing from 39.0 yr to 15.8 yr) heterogeneity. Second, we tested hypothetical vaccines that conferred a mean duration of protection of 5 yr. As expected, estimation performance substantially degraded in this case, but on the whole, the results were consistent with those for slow-waning vaccines that confer a mean duration of protection of 2 yr. Specifically, assuming the degree of variability to be known *a priori*, accurate and relatively precise estimation with 1 yr of data was possible for high-heterogeneity vaccines (electronic supplementary material, figure S9). By contrast, vaccines with lower heterogeneity led to practical non-identifiabilities and imprecise estimates, though a lower bound could again be derived in every scenario (range of 99% CI lower bounds: 1.1 to 3.8 yr). For low-heterogeneity vaccines, it was not possible to estimate the variability in the duration of protection in any scenario (electronic supplementary material, figure S10, top panel). This lack of identifiability was also typical for high-heterogeneity vaccines, although the full duration of protection could be estimated in some scenarios (2/10 scenarios; electronic supplementary material, figure S10, bottom panel). Third, we assessed the robustness of our results to the inclusion of one additional estimated parameter, either the relative infectiousness (*θ*) or the reporting probability (*ρ*_S_) of subclinical infections. For slow-waning vaccines and a study period of 1 yr, in either case the extra parameter was estimated close to its true value and did not correlate strongly with the average duration of protection, which thus exhibited similar ranges of uncertainty (electronic supplementary material, figure S11).

## 4. Discussion

In this simulation study of the post-vaccination dynamics of an emerging pathogen, we aimed to determine the amount of daily, age-specific case report incidence data required for transmission models to appropriately estimate the duration of vaccine protection. Focusing on SARS-CoV-2 in Germany as a case study, we tested a range of vaccines with different distributions of the duration of protection, specified by their mean (tested values: 0.85 yr (rapid-waning vaccines) and 2 yr (slow-waning vaccines)) and their coefficient of variation (tested values: 50% (low-heterogeneity vaccines), 71%, and 100% (high-heterogeneity vaccines)). Across the vaccines tested, rapid waning and high heterogeneity permitted complete identification of the duration of protection. By contrast, slow waning and low heterogeneity allowed only estimation of the fraction of vaccinees with rapid loss of immunity. These results suggest that even a limited amount of epidemiological data can provide some information on the duration of vaccine protection. In addition, they show that higher vaccine heterogeneity can facilitate estimation of this duration and thus highlight the importance of considering this heterogeneity in epidemiological models of vaccines.

The main, robust finding of our study was that heterogeneity can facilitate estimation of the duration of vaccine protection. This result may be explained by the impact of heterogeneity on the shape of the distribution of vaccine protection durability. Specifically, even when the mean duration of protection or the proportion of vaccinees losing immunity during the study period are kept constant, more heterogeneous vaccines cause faster accumulation of susceptible individuals who lose vaccine immunity (electronic supplementary material, figure  S4). Hence, more heterogeneous vaccines may lead to higher infection incidence and therefore leave stronger dynamical footprints in epidemiological time series (electronic supplementary material, figures S6, S7). Because this effect of heterogeneity is generic, our results may apply to vaccines against pathogens beyond SARS-CoV-2. Importantly, these observations are specific to the type of heterogeneity considered here (that is, heterogeneity of vaccine duration of protection), as other studies demonstrated that heterogeneity of other vaccine properties—like susceptibility to infection—acts to reduce the burden of infection [4,6]. Finally, even though we identified vaccine heterogeneity as a predictor of identifiability, substantial variability in estimation performance frequently remained across the epidemiological scenarios. This demonstrates the existence of other predictors related to temporal variations in transmission, which were not examined here but could be the object of further research.

More generally, in keeping with previous modelling studies [4–6], our results highlight the need to pay careful attention to heterogeneity of vaccine protection when modelling the population-level impact of vaccines. In practice, we recommend testing multiple models with different levels of heterogeneity and, when this heterogeneity cannot be identified from data, using ensemble predictions to convey the corresponding uncertainty. As the exponential distribution (with high heterogeneity) is a standard assumption in the modelling literature, failure to consider alternative assumptions may lead to false confidence in model accuracy and precision. In that case, even though some aspects of the duration of protection (like the fraction with short-term immunity) could still be well estimated, the model's long-term predictions may suffer from severe bias.

Our study has important limitations, which result from our deliberate choice to use a simple, yet sufficiently general model of vaccination. First, we did not consider the possibility of a concomitant roll-out of multiple vaccines, yet a common situation in the field. Our simple model could still be applied to multiple vaccines deemed to confer comparable immunity (such as mRNA vaccines for COVID-19), but will require extension otherwise, for example by considering multiple vaccinated compartments with distinct vaccine parameters. However, this would require additional data on vaccine-specific coverage data and external evidence from vaccine studies to inform the fixed value of vaccine properties other than the duration of protection. Second, the high mutation rate of some emerging pathogens (like SARS-CoV-2  and other RNA viruses) may cause the emergence of vaccine-escape variants that progressively modify the characteristics of vaccine protection. In this case, our assumption of fixed vaccine properties would need to be revised; we believe, however, it is reasonable because of the short time period (1 yr) considered in our study. Third, we assumed that post-vaccine infections (i.e. infections after immunity had waned) in vaccinees had the same characteristics as primary infections in immunologically naive individuals. In reality, more complex immunological mechanisms may be at play, such that partial protection against infection or transmission is maintained via recall B or T cell memory response, even after disappearance of residual sterilizing immunity (effected, for example, by neutralizing antibodies [52]). Differences between post-vaccine and primary infections—e.g. in terms of infectiousness, duration, and severity—may sensitively shape the long-term dynamics of an emerging pathogen like SARS-CoV-2 [53] and could be examined with specific vaccine studies, for example studies designed to estimate vaccine effectiveness on infectiousness or on disease progression in vaccinated breakthrough cases [2,54]. Such estimates, already available for COVID-19 vaccines [55], could be readily integrated into more detailed epidemiological models. Fourth, we considered only parametric uncertainty, assuming a true underlying model of vaccine protection and thus ignoring the inductive uncertainty associated with the formulation of this model itself [56]. As noted above, however, our model's underlying assumptions—that is, imperfect and waning vaccine immunity—are largely generic and may be valid for multiple classes of vaccines. Fifth, we used only deterministic process models for statistical inference, because fully stochastic age-structured models are much more computationally intensive to estimate, typically requiring particle filtering and extensive simulations [44]. In practical applications limited to a few datasets, however, fully stochastic models are preferable, as they offer more flexibility and biological realism and also permit better quantification of forecasting uncertainty [57]. Sixth, we did not attempt to assess the consistency of our estimation procedure, even though an earlier simulation study that used a comparable model and identical inference techniques found sizable differences between the nominal coverage and the achieved coverage [57]. Finally, for simplicity and interpretability, we assumed that the duration of vaccine protection could be modelled by a simple statistical distribution, fully specified by its first two moments (mean and variance). Nevertheless, more complex distributions may be required to capture the heterogeneity of vaccine protection.

In conclusion, our study provides evidence that only a short period of time may be required to estimate some characteristics of vaccine immunity, even in the face of uncertainties about temporal variations in transmission of an emerging pathogen. Echoing earlier findings [4], our results also highlight the need to accurately quantify heterogeneity of vaccine protection to predict the impact of mass immunization. More generally, a more systematic examination of the consequences of heterogeneity—the norm in biology [58]—may be warranted, not only for the study of COVID-19 vaccines but also of other vaccines.

## Data Availability

All R programming codes are freely available from Edmond, the Open Data Repository of the Max Planck Society: https://doi.org/10.17617/3.AWRHE8. The data are provided in electronic supplementary material [59].

## References

[1] Keeling MJ, Rohani P. 2008 Modeling infectious diseases in humans and animals. Princeton, NJ: Princeton University Press.

[2] Halloran ME, Longini IM, Struchiner CJ, Longini IM. 2010 Design and analysis of vaccine studies, vol. 18. Berlin, Germany: Springer.

[3] Magpantay FMG, Riolo MA, Domenech de Cellès M, King AA, Rohani P. 2014 Epidemiological consequences of imperfect vaccines for immunizing infections. SIAM J. Appl. Math. **74**, 1810-1830. (10.1137/140956695) 25878365 PMC4394665

[4] Gomes MGM, Lipsitch M, Wargo AR, Kurath G, Rebelo C, Medley GF, Coutinho A. 2014 A missing dimension in measures of vaccination impacts. PLoS Pathog. **10**, e1003849. (10.1371/journal.ppat.1003849) 24603721 PMC3946326

[5] Woolthuis RG, Wallinga J, Boven Mv. 2017 Variation in loss of immunity shapes influenza epidemics and the impact of vaccination. BMC Infect. Dis. **17**, 632. (10.1186/s12879-017-2716-y) 28927373 PMC5606000

[6] White MT, Griffin JT, Drakeley CJ, Ghani AC. 2010 Heterogeneity in malaria exposure and vaccine response: implications for the interpretation of vaccine efficacy trials. Malar. J. **9**, 82. (10.1186/1475-2875-9-82) 20331863 PMC2851701

[7] Antia A, Ahmed H, Handel A, Carlson NE, Amanna IJ, Antia R, Slifka M. 2018 Heterogeneity and longevity of antibody memory to viruses and vaccines. PLoS Biol. **16**, e2006601. (10.1371/journal.pbio.2006601) 30096134 PMC6105026

[8] Pollard AJ, Bijker EM. 2021 A guide to vaccinology: from basic principles to new developments. Nat. Rev. Immunol. **21**, 83-100. (10.1038/s41577-020-00479-7) 33353987 PMC7754704

[9] Goel RR et al. 2021 mRNA vaccines induce durable immune memory to SARS-CoV-2 and variants of concern. Science **374**, eabm0829. (10.1126/science.abm0829) PMC9284784 34648302

[10] Crowcroft NS et al. 2021 A call for caution in use of pertussis vaccine effectiveness studies to estimate waning immunity: a Canadian Immunization Research Network Study. Clin. Infect. Dis. **73**, 83-90. (10.1093/cid/ciaa518) 32384142 PMC8246842

[11] Smith PG, Rodrigues LC, Fine PE. 1984 Assessment of the protective efficacy of vaccines against common diseases using case-control and cohort studies. Int. J. Epidemiol. **13**, 87-93. (10.1093/ije/13.1.87) 6698708

[12] Domenech de Cellès M, Rohani P, King AA. 2019 Duration of immunity and effectiveness of diphtheria-tetanus-acellular pertussis vaccines in children. JAMA Pediatr. **173**, 588-594. (10.1001/jamapediatrics.2019.0711) 31009031 PMC6547082

[13] Lavine JS, Rohani P. 2012 Resolving pertussis immunity and vaccine effectiveness using incidence time series. Expert Rev. Vaccines **11**, 1319-1329. (10.1586/erv.12.109) 23249232 PMC3595187

[14] Domenech de Cellès M, King AA, Rohani P. 2019 Commentary: resolving pertussis resurgence and vaccine immunity using mathematical transmission models. Hum. Vaccin. Immunother. **15**, 683-686. (10.1080/21645515.2018.1549432) 30457424 PMC6988877

[15] Halloran ME et al. 2017 Simulations for designing and interpreting intervention trials in infectious diseases. BMC Med. **15**, 223. (10.1186/s12916-017-0985-3) 29287587 PMC5747936

[16] Pitzer VE, Basta NE. 2012 Linking data and models: the importance of statistical analyses to inform models for the transmission dynamics of infections. Epidemiology **23**, 520-522. (10.1097/EDE.0b013e31825902ab) 22659545 PMC3399508

[17] Blackwood JC, Cummings DAT, Broutin H, Iamsirithaworn S, Rohani P. 2013 Deciphering the impacts of vaccination and immunity on pertussis epidemiology in Thailand. Proc. Natl Acad. Sci. USA **110**, 9595-9600. (10.1073/pnas.1220908110) 23690587 PMC3677483

[18] Argante L, Tizzoni M, Medini D. 2016 Fast and accurate dynamic estimation of field effectiveness of meningococcal vaccines. BMC Med. **14**, 98. (10.1186/s12916-016-0642-2) 27363534 PMC4929770

[19] Domenech de Cellès M, Magpantay FMG, King AA, Rohani P. 2018 The impact of past vaccination coverage and immunity on pertussis resurgence. Sci. Transl. Med. **10**, eaaj1748. (10.1126/scitranslmed.aaj1748) 29593103 PMC6063734

[20] Lewnard JA, Grad YH. 2018 Vaccine waning and mumps re-emergence in the United States. Sci. Transl. Med. **10**, eaao5945. (10.1126/scitranslmed.aao5945) 29563321 PMC5899613

[21] Davies NG et al. 2021 Estimated transmissibility and impact of SARS-CoV-2 lineage B.1.1.7 in England. Science **372**, eabg3055. (10.1126/science.abg3055) 33658326 PMC8128288

[22] Davies NG, Klepac P, Liu Y, Prem K, Jit M, CMMID COVID-19 working group. 2020 Age-dependent effects in the transmission and control of COVID-19 epidemics. Nat. Med. **26**, 1205-1211. (10.1038/s41591-020-0962-9) 32546824

[23] Li R, Pei S, Chen B, Song Y, Zhang T, Yang W, Shaman J. 2020 Substantial undocumented infection facilitates the rapid dissemination of novel coronavirus (SARS-CoV-2). Science **368**, 489-493. (10.1126/science.abb3221) 32179701 PMC7164387

[24] Mossong J et al. 2008 Social contacts and mixing patterns relevant to the spread of infectious diseases. PLoS Med. **5**, e74. (10.1371/journal.pmed.0050074) 18366252 PMC2270306

[25] Rohani P, Zhong X, King AA. 2010 Contact network structure explains the changing epidemiology of pertussis. Science **330**, 982-985. (10.1126/science.1194134) 21071671

[26] Campbell F et al. 2021 Increased transmissibility and global spread of SARS-CoV-2 variants of concern as at June 2021. Eurosurveillance **26**, 2100509. (10.2807/1560-7917.ES.2021.26.24.2100509) 34142653 PMC8212592

[27] Abbott S, Bennett C, Hickson J, Allen J, Sherratt K, Funk S. 2020 National and subnational estimates of the Covid 19 reproduction number (R) for Germany based on test results. Harvard Dataverse. (10.7910/DVN/LNMJYJ)

[28] Arora RK et al. 2021 SeroTracker: a global SARS-CoV-2 seroprevalence dashboard. Lancet Infect. Dis. **21**, e75-e76. (10.1016/S1473-3099(20)30631-9) 32763195 PMC7402646

[29] Walsh EE et al. 2020 Safety and immunogenicity of two RNA-based COVID-19 vaccine candidates. N. Engl. J. Med. **383**, 2439-2450. (10.1056/NEJMoa2027906) 33053279 PMC7583697

[30] Anderson EJ et al. 2020 Safety and immunogenicity of SARS-CoV-2 mRNA-1273 vaccine in older adults. N. Engl. J. Med. **383**, 2427-2438. (10.1056/NEJMoa2028436) 32991794 PMC7556339

[31] Jackson LA et al. 2020 An mRNA vaccine against SARS-CoV-2: preliminary report. N. Engl. J. Med. **383**, 1920-1931. (10.1056/NEJMoa2022483) 32663912 PMC7377258

[32] Folegatti PM et al. 2020 Safety and immunogenicity of the ChAdOx1 nCoV-19 vaccine against SARS-CoV-2: a preliminary report of a phase 1/2, single-blind, randomised controlled trial. Lancet **396**, 467-478. (10.1016/S0140-6736(20)31604-4) 32702298 PMC7445431

[33] Lloyd AL. 2001 Destabilization of epidemic models with the inclusion of realistic distributions of infectious periods. Proc. R. Soc. Lond. B **268**, 985-993. (10.1098/rspb.2001.1599) PMC1088698 11370974

[34] Arinaminpathy N, Kim IK, Gargiullo P, Haber M, Foppa IM, Gambhir M, Bresee J. 2017 Estimating direct and indirect protective effect of influenza vaccination in the United States. Am. J. Epidemiol. **186**, 92-100. (10.1093/aje/kwx037) 28369163 PMC5860220

[35] Russell TW et al. 2020 Reconstructing the early global dynamics of under-ascertained COVID-19 cases and infections. BMC Med. **18**, 332. (10.1186/s12916-020-01790-9) 33087179 PMC7577796

[36] Santos-Hövener C et al. 2020 Serology- and PCR-based cumulative incidence of SARS-CoV-2 infection in adults in a successfully contained early hotspot (comolo study), Germany, May to June 2020. Eurosurveillance **25**, 2001752. (10.2807/1560-7917.ES.2020.25.47.2001752) 33243353 PMC7693167

[37] European Centre for Disease Prevention and Control. 2021 Overview of COVID-19 vaccination strategies and vaccine deployment plans in the EU/EEA and the UK. European Centre for Disease Prevention and Control.

[38] Robert Koch Institute. 2021 *COVID-19-Impfungen in Deutschland*. See https://github.com/robert-koch-institut/COVID-19-Impfungen_in_Deutschland (accessed 1 September 2021).

[39] Barouch DH et al. 2021 Durable humoral and cellular immune responses 8 months after Ad26.COV2.S vaccination. N. Engl. J. Med. **385**, 951-953. (10.1056/NEJMc2108829) 34260834 PMC8314733

[40] Doria-Rose N et al. 2021 Antibody persistence through 6 months after the second dose of mRNA-1273 vaccine for COVID-19. N. Engl. J. Med. **384**, 2259-2261. (10.1056/NEJMc2103916) 33822494 PMC8524784

[41] Polack FP et al. 2020 Safety and efficacy of the BNT162b2 mRNA COVID-19 vaccine. N. Engl. J. Med. **383**, 2603-2615. (10.1056/NEJMoa2034577) 33301246 PMC7745181

[42] Baden LR et al. 2021 Efficacy and safety of the mRNA-1273 SARS-CoV-2 vaccine. N. Engl. J. Med. **384**, 403-416. (10.1056/NEJMoa2035389) 33378609 PMC7787219

[43] Dagan N et al. 2021 BNT162b2 mRNA COVID-19 vaccine in a nationwide mass vaccination setting. N. Engl. J. Med. **384**, 1412-1423. (10.1056/NEJMoa2101765) 33626250 PMC7944975

[44] King AA, Nguyen D, Ionides EL. 2016 Statistical inference for partially observed markov processes via the R package pomp. J. Stat. Softw. **69**, 1-43. (10.18637/jss.v069.i12)

[45] Raue A, Kreutz C, Maiwald T, Bachmann J, Schilling M, Klingmüller U, Timmer J. 2009 Structural and practical identifiability analysis of partially observed dynamical models by exploiting the profile likelihood. Bioinformatics **25**, 1923-1929. (10.1093/bioinformatics/btp358) 19505944

[46] King AA et al. 2021 pomp: statistical inference for partially observed Markov processes. R package, version 2.7. See https://kingaa.github.io/pomp/.

[47] R Core Team. 2020 R: a language and environment for statistical computing. Vienna, Austria: R Foundation for Statistical Computing. See https://www.R-project.org/.

[48] Funk S. 2021 socialmixr: social mixing matrices for infectious disease modelling. R package version 0.1.8. See https://CRAN.R-project.org/package=socialmixr.

[49] Ushey K. 2021 renv: project environments. R package version 0.12.5. See https://CRAN.R-project.org/package=renv.

[50] Ramasamy MN et al. 2021 Safety and immunogenicity of ChAdOx1 nCoV-19 vaccine administered in a prime-boost regimen in young and old adults (COV002): a single-blind, randomised, controlled, phase 2/3 trial. Lancet **396**, 1979-1993. (10.1016/S0140-6736(20)32466-1) 33220855 PMC7674972

[51] Voysey M et al. 2021 Safety and efficacy of the ChAdOx1 nCoV-19 vaccine (AZD1222) against SARS-CoV-2: an interim analysis of four randomised controlled trials in Brazil, South Africa, and the UK. Lancet **397**, 99-111. (10.1016/S0140-6736(20)32661-1) 33306989 PMC7723445

[52] Cromer D, Juno JA, Khoury D, Reynaldi A, Wheatley AK, Kent SJ, Davenport MP. 2021 Prospects for durable immune control of SARS-CoV-2 and prevention of reinfection. Nat. Rev. Immunol. **21**, 395-404. (10.1038/s41577-021-00550-x) 33927374 PMC8082486

[53] Saad-Roy CM et al. 2020 Immune life history, vaccination, and the dynamics of SARS-CoV-2 over the next 5 years. Science **370**, 811-818. (10.1126/science.abd7343) 32958581 PMC7857410

[54] Halloran ME, Struchiner CJ, Longini Jr IM. 1997 Study designs for evaluating different efficacy and effectiveness aspects of vaccines. Am. J. Epidemiol. **146**, 789-803. (10.1093/oxfordjournals.aje.a009196) 9384199

[55] de Gier B, Andeweg S, Backer JA, RIVM COVID-19 surveillance and epidemiology team, Hahné SJ, van den Hof S et al. 2021 Vaccine effectiveness against SARS-CoV-2 transmission to household contacts during dominance of Delta variant (B.1.617.2), the Netherlands, August to September 2021. Eurosurveillance **26**, 2100977. (10.1101/2021.10.14.21264959) 34738514 PMC8569927

[56] Pawitan Y. 2001 In all likelihood: statistical modelling and inference using likelihood. Oxford, UK: Oxford University Press.

[57] King AA, Domenech de Cellès M, Magpantay FMG, Rohani P. 2015 Avoidable errors in the modelling of outbreaks of emerging pathogens, with special reference to Ebola. Proc. Biol. Sci. **282**, 20150347. (10.1098/rspb.2015.0347) 25833863 PMC4426634

[58] Schwartz D. 1994 Le jeu de la science et du hasard: la statistique et le vivant. Paris, France: Flammarion. [In French]

[59] Domenech de Cellès M, Wong A, Barrero Guevara LA, Rohani P. 2022 Immunological heterogeneity informs estimation of the durability of vaccine protection. *Figshare*. (10.6084/m9.figshare.c.5973577) PMC9131131 35611620

